# TriHex 2.0—Advancing Skin Health Science and the TriHex Technology

**DOI:** 10.1111/jocd.16690

**Published:** 2024-12-11

**Authors:** Alan D. Widgerow, Mary E. Ziegler, Faiza Shafiq

**Affiliations:** ^1^ Division Chief Research, Professor Plastic Surgery Center for Tissue Engineering University of California Irvine California USA; ^2^ Alastin a Galderma Company Fort Worth Texas USA

**Keywords:** elastin, extracellular matrix, hyaluronic acid, peptides, topical agent, topical anti‐aging

## Abstract

**Background:**

The original TriHex combination—Tripeptide‐1 and Hexapeptide‐12 (TriHex) encompasses a peptide combination selected for its ability to modulate the extracellular matrix (ECM) by progressively eliminating clumped collagen and elastin fragments and then stimulating replacement with new collagen and elastin. Incorporation of a proprietary, patent‐pending Octapeptide‐45 (Octa) to the TriHex original provides potential for added benefit based on the peptide's capacity to stimulate hyaluronic acid (HA) and its anticipated added benefit in wound healing. This is named TriHex 2.0 in the paper.

**Materials and Methods:**

A full‐scale validation process was structured to assess Octa synergy with TriHex using an ex vivo model, assessing ECM changes histologically in relation to elastin, HA and basement membrane components. In addition, gene expression studies were undertaken, including bulk and single cell sequencing analysis to assess the particular changes that occurred by adding Octa to the TriHex. Following the gene expression analysis, a further round of ex vivo studies was conducted to assess protein expression of the defined differentially expressed genes using histological staining.

**Results:**

Octa synergized with TriHex as demonstrated by significantly upregulated genes (*p* < 0.05) affecting the ECM and basement membrane. A histological assessment using the ex vivo model demonstrated tropoelastin intensity significantly increasing with TriHex (43%) and 2.0 (42%) (*p* < 0.05 for both) compared to untreated explants. HA levels (CD44 intensity) significantly increased with TriHex (69%; *p* < 0.01), while TriHex 2.0 demonstrated HA levels 160% greater (*p* < 0.001) than the untreated tissue. Single cell sequencing identified a gene expression profile upregulation relating to ECM modulation and wound healing in both TriHex and 2.0, but TriHex 2.0 showed additional activities in basement membrane physiology, stem cell recruitment, and protection of fibroblasts against cellular senescence.

**Conclusion:**

The addition of Octapeptide‐45 to TriHex technology in the form of TriHex 2.0 is a significant advance to TriHex technology science. Both forms demonstrate ECM remodeling and positive wound healing, but supplementary benefits are evident including increased elastin and hyaluronic acid stimulation, added effects on the basement membrane, additional wound healing capacity in basal keratinocytes and anti‐senescent effects in fibroblasts. This is helpful for pre‐conditioning of the skin prior to procedures and post procedure related to additional ECM remodeling, wound healing advantages, senescent cell targeting and DEJ strengthening. Clinical studies to follow.

## 1 Background

Daily living takes its toll on the skin as much as any other organ, in some ways more so than other organs. This relates to the fact that exposed skin is subjected to daily photodamage that cumulatively results in fragmentation of collagen and elastin and gradual restructuring and disorganization of the extracellular matrix (ECM) of the dermis. Add to this, the intrinsic changes that occur with aging and the manifestations of the damage become obvious—loss of elasticity, fine lines, pigmentation changes and limited regeneration capacity of cells like the fibroblasts which become senescent in this dysfunctional ECM [1].

The original TriHex combination—Tripeptide‐1 and Hexapeptide‐12 (TriHex) encompasses a peptide combination selected for its ability to target the ECM changes described above. In a sequence of experiments and biopsies, it was demonstrated that these peptides progressively break down the clumped collagen/gelatin and elastin fragments and then stimulate replacement with new collagen and elastin, effectively recycling the ECM [2, 3, 4, 5]. This concept has been used not only with skin health maintenance objectives [6] but also as a major indication for skin bed preparation prior to varied aesthetic procedures [4, 5, 7, 8, 9, 10, 11]. In addition, the anhydrous gel (Regenerating Skin Nectar with TriHex Technology) has been used following skin laser procedures to optimize healing, reducing downtime and providing symptomatic relief (less redness, exudate, pain, itching, etc.) following invasive resurfacing procedures [7, 8, 10]. In addition, the Restorative Skin Complex with TriHex Technology product (anti‐aging line) uses the same TriHex peptides and additional actives as anti‐aging maintenance by recycling the ECM and stimulating new collagen and elastin production [11].

As such, over the past 8 years the TriHex technology incorporated in Regenerating Skin Nectar with TriHex Technology has been used in hundreds of thousands of cases with an extremely low adverse event rate (data on file). Based on the success over this time period, a new scientific endeavor was undertaken to advance the TriHex technology concept to its next level—TriHex 2.0.

Incorporation of a proprietary, patent‐pending Octapeptide‐45 (Octa) to the TriHex original made logical sense based on the properties discovered in this proprietary in‐house designed peptide [12, 13]. The potential for added benefit was based on the peptide's capacity for hyaluronic acid stimulation and its anticipated added benefit in wound healing [14]. However, a full‐scale validation process was structured to assess synergy with TriHex using an ex vivo model, assessing ECM changes histologically in relation to elastin and other components, and gene expression single cell analysis assessing the particular changes that occurred by adding Octa. These findings are highlighted below.

## 2 Materials and Methods

### 2.1 Cell Line and Peptide Combination Treatments

Human adult dermal fibroblasts were purchased from ZenBio (Durham, NC) and cultured in fibroblast media (ZenBio). The cells were maintained at 37°C in a 5% CO_2_ incubator. For the treatments, the fibroblasts were seeded into 48‐well plates. After 48 h, the cells were exposed to the following compounds (all peptides at 20 mg/mL) for 24 h.

1. TriHex
2. Octa
3. TriHex + Octa

One group was left untreated for comparison purposes. After 24 h, the RNA was extracted, and RNA sequencing was performed to reveal differential gene expression.

#### 2.1.1 RNA Lysate Preparation

After 24 h of compound exposure, the media was removed, and the cells were washed once with PBS. Then, lysates were generated using 100 μL of RNA Lysis Buffer (Takara Bio Cat Num 635013, “10× RNA lysis buffer”, diluted to 1×) in RNAse‐free microcentrifuge tubes. The samples were immediately frozen at −20°C.

#### 2.1.2 Bulk RNA Sequencing

All the RNA samples were shipped frozen on dry ice to MedGenome (Foster City, CA) for RNA extraction, library construction, and sequencing to 25 M paired‐end 100 bp reads per sample. Differentially expressed genes were identified and pathway enrichment was assessed using the Reactome Pathway database.

### 2.2 Ex Vivo Culture—Elastogenesis and Hyaluronic Acid (HA) Stimulation

All human discarded facial tissue from facelifts was obtained under an IRB approved study. Tissue was kept chilled until processing for culture (between 16 and 24 h post‐removal) and processed for use within 24 h of surgery. The tissue was defatted and cut into a diameter of approximately 5 mm and placed in a transwell insert with cell culture media. The medium was DMEM/F12 containing 1.88 μM CaCl_2_, 2% fetal bovine serum, 50 μM adenine, 0.02 nM tri‐iodothyronine, 1% ITS‐X (insulin, transferrin, selenium mix), 1% glutagro (a glutamine equivalent), 1% pen/strep, and 1% gentamycin. The medium was changed every day. The human tissue explants were equilibrated for 3 days, and then, the treatments were applied. The treatments (all peptides at 20 μg/mL) were TriHex, Octa, TriHex + Octa, and no treatment. The treatment combinations were based on the in vitro RNA‐seq data, which identified TriHex + Octa as a potential ECM regulator. The treatments were administered every day for 7 days.

After the treatments, the skin explants were fixed in formalin and embedded in paraffin following standard procedures. The tissue was sectioned, and the sections were deparaffinized and rehydrated following standard protocols. The sections were prepared for immunofluorescence to detect elastin using an anti‐tropoelastin antibody (Elastin, Owensville, MO) and microfibril‐associated protein 4 (MFAP4) using and anti‐MFAP4 antibody (Thermo Fisher, Waltham, MA) and CD44 staining for HA. The appropriate fluorescent secondary antibodies were used for detection by fluorescence microscopy. Images were obtained and analyzed using ImageJ software. Data were obtained from a minimum of three donor skin samples and replicates or triplicates for each treatment.

### 2.3 Ex Vivo Culture—Single‐Cell RNA Sequencing

Skin was prepared for ex vivo culture as before obtaining 5 mm skin patches. Three patient samples were obtained and replicates were tested. Three skin patches were treated with TriHex 1.0 full formulation (commercial product, Regenerating Skin Nectar with TriHex Technology), which was applied every 24 h for 7 days. Three skin patches were treated with TriHex 2.0 full formulation (TriHex 2.0, TriHex + Octa), which was also applied every 24 h for 7 days. The remaining three skin patches were maintained in media as the untreated control. Cells were dissociated to single cells using Miltenyi Whole Skin Dissociation Kit (#130–101‐540), and then washed in PBS and fixed using the 10× Genomics Chromium Next GEM Single Cell Fixed RNA Sample Preparation Kit (PN1000414). They were then stored at −80°C as per kit instructions. Approximately 6000 cells per sample were run in the FLEX single cell assay as per 10× Genomics Chromium Fixed RNA Profiling Reagent Kits User Guide (CG000527). The libraries were then sequenced to about 50k reads per cell and mapped in CellRanger.

### 2.4 Ex Vivo Staining Based on Single Cell Sequencing Results

The investigation outlined in section three was repeated—sections were prepared for immunofluorescence but this time to detect proteins related to gene upregulation identified in section three that were particular to TriHex 2.0. Thus, antibodies and appropriate fluorescent secondary antibodies were used to detect CCN1, ColIV, CXCL12, Laminin 5, and Lumican by fluorescence microscopy. Images were obtained and analyzed using ImageJ software. Data were obtained from at least three donor skin samples with replicates for each treatment.

## 3 Results

### 3.1 Octa Synergizes With TriHex to Induce Gene Upregulation

**FIGURE 1:**
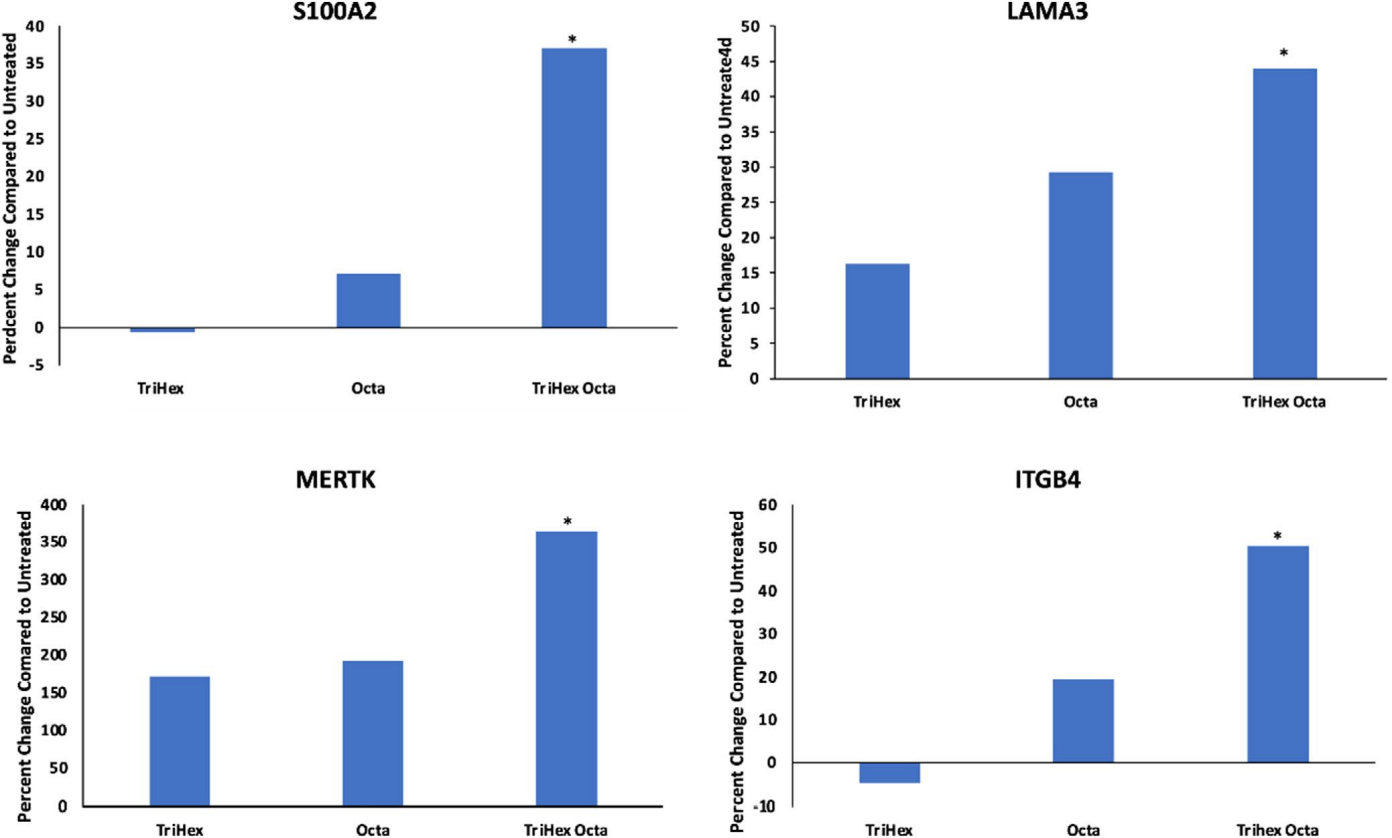
TriHex + octa gene expression synergy in fibroblasts. Human dermal fibroblasts were cultured, left untreated, or treated with TriHex, Octa, or TriHex + Octa. RNA was extracted and sequenced. The differential gene expression results revealed a synergy for the genes presented when TriHex was combined with Octa. The data are presented as the percent change relative to the untreated cells. *p < 0.05.

Human dermal fibroblasts were cultured and treated with: TriHex, Octa, or TriHex + Octa. Bulk RNA sequencing revealed four genes that showed synergistic significant upregulated gene expression in the TriHex + Octa group (Figure 1).

#### 3.1.1 Gene Information

1. S100A2 is S100 Calcium Binding Protein A2. It is a secreted matrisome‐associated protein that is expressed in the dermis. S100A2 activates protein phosphatase 5 (PP5) in a calcium‐dependent manner. PP5 is a serine/threonine phosphatase involved in oxidative stress responses [15].
2. LAMA3 is Laminin, alpha 3 that encodes the alpha‐3 chain of laminin 5, the major link between the epidermal basal cells and the papillary dermis, providing stable attachment of the epidermis to the dermis. Laminin 5 also aids in assembly of basement membranes and may enhance the recovery of damaged skin [16].
3. MERTK is Tyrosine‐protein kinase that transduces signals from the extracellular matrix into the cytoplasm by binding to several ligands involved in processes such as macrophage clearance of apoptotic cells, platelet aggregation and cytoskeleton reorganization [17].
4. ITGB4 is integrin beta 4 and partnered with integrin alpha 6, is the receptor for laminin involved in strengthening and stabilizing the skin as a component of hemidesmosomes in the epidermis [18].
5. These upregulated genes provided clues that increased activity in the basement membrane and ECM were likely happening.

### 3.2 Ex Vivo Elastogenesis Assessment

**FIGURE 2:**
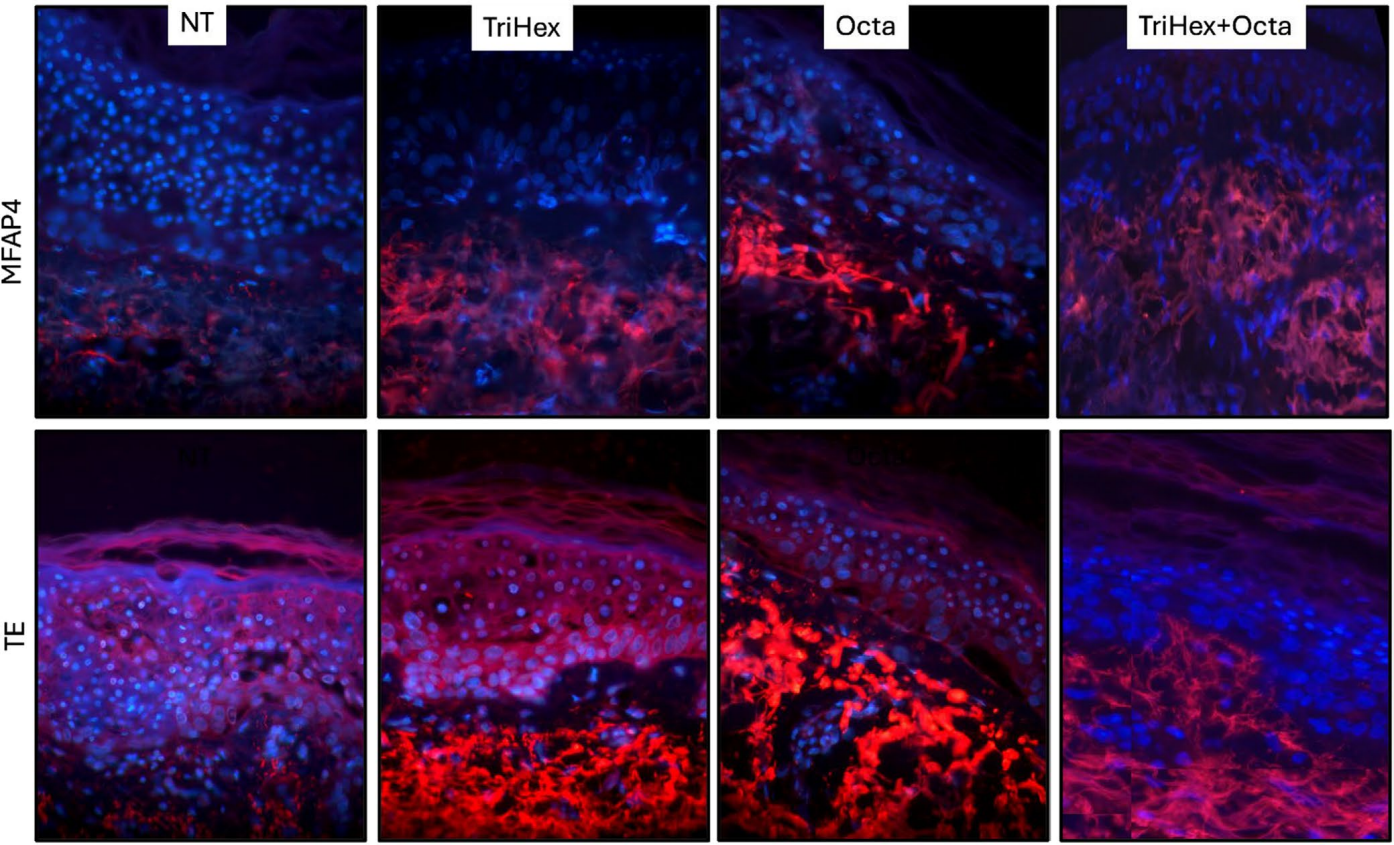
Elastogenesis induction. Skin samples were cultured using the established ex vivo model. They were left untreated or treated with TriHex, Octa, or TriHex + Octa. After 7 days of treatment, the tissue sections were processed to detect markers of elastogenesis. The top panels depict microfibrillar associated protein 4 (MFAP4) in red and the bottom panels show tropoelastin (TE) in red. Nuclei are depicted by DAPI and shown in blue.

To detect the production of new elastin after the treatment, skin samples were cultured using the established ex vivo model. TTE and MFAP4 were detected by antibody‐based staining. TE is a soluble precursor of elastin. The deposition of TE gives rise to a central core of elastin that is surrounded by fibrillin‐rich microfibrils, including MFAP4 (Figure 2).

The combination of the 3 peptides depicted in the column on the extreme right appear to have an increased distribution and infiltration of tropoelastin and MFAP4 into papillary dermis and DEJ. In addition, the tropoelastin fibers depicted in the same column (bottom R) appear long, flowing and intact resembling healthy tropoelastin.

**FIGURE 3:**
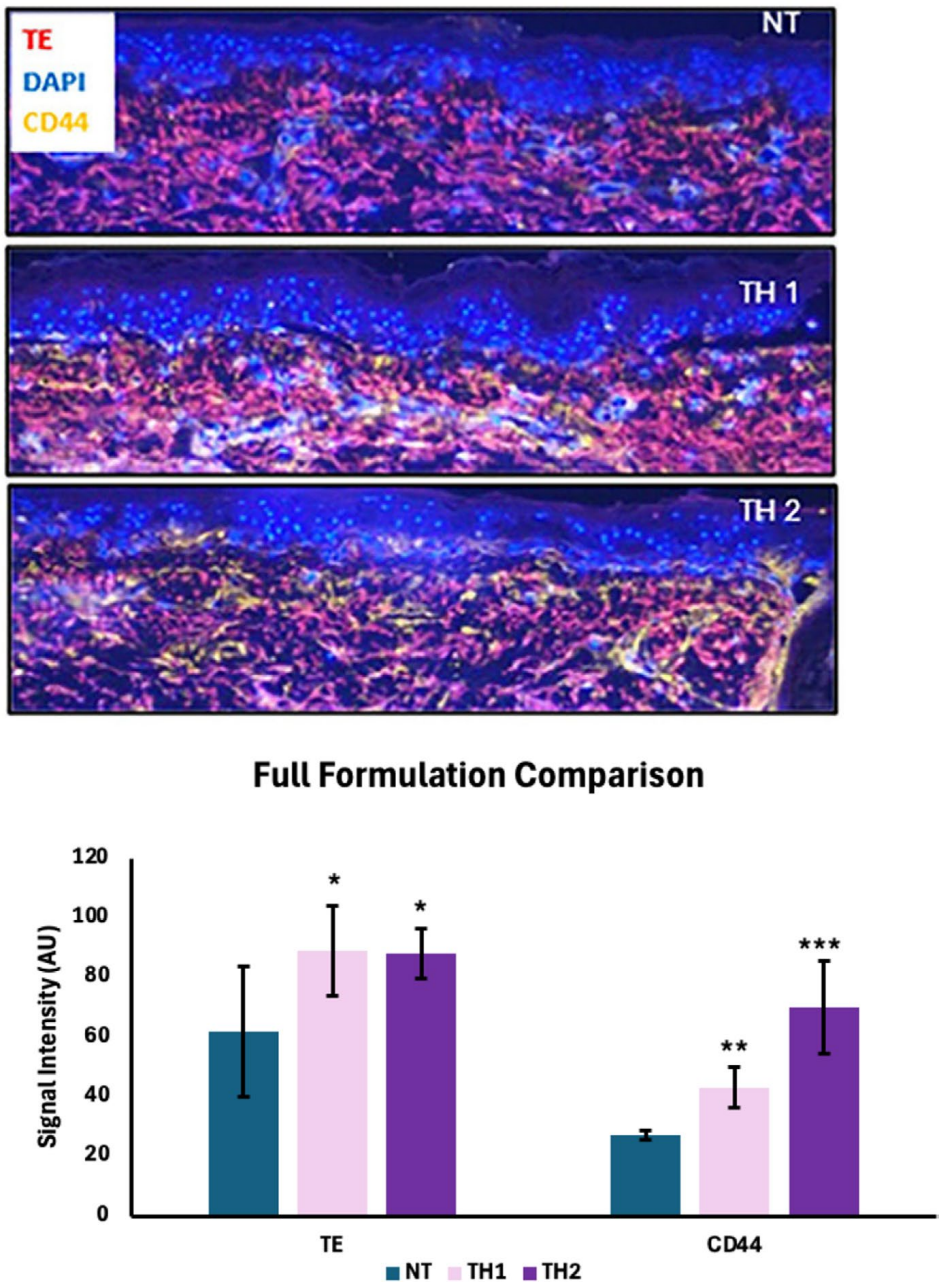
TriHex 2.0 significantly increases HA production. Skin samples were cultured using the established ex vivo model. They were left untreated or treated with TriHex 1.0 (TH1) or TriHex 2.0 (TH2). After 7 days of treatment, the tissue sections were processed for immunostaining. Tropoelastin (TE) is red, CD44 (HA) is yellow, and the nuclei are shown in blue. Images were quantified using ImageJ to assess the intensity of the markers. These values (arbitrary units [AU]) are presented as the mean ± SD of 4–5 samples, and significance is relative to the non‐treated (NT) samples (*p < 0.05; **p < 0.01; ***p < 0.001).

To demonstrate the added effect of Octa as a full formulation, TriHex 1.0 full formulation (TH1) was compared to TriHex 2.0 full formulation (TH2) using the ex vivo model. The expression of TE was significantly enhanced for TH1 and TH2 (*p* < 0.05 for both). CD44, as a marker for HA, was significantly upregulated by TH1 and TH2‐treated samples demonstrated an even greater increase (Figure 3).

### 3.3 Ex Vivo Single Cell Sequencing Results—TriHex 1.0 and 2.0—Analysis of Genes Upregulated in Keratinocytes, Basal Keratinocytes and Fibroblast Cells

#### 3.3.1 Keratinocytes

The keratinocyte population revealed 353 genes upregulated by TriHex 1.0 and 292 by TriHex 2.0. There were 126 genes common to TriHex 1.0 and 2.0. Some key genes are described here common to both groups. Follistatin stimulates cellular proliferation and aids in epidermal wound healing [19]. In addition, it has been used in the treatment of radiation‐induced fibrosis [19, 20]. HAS three is involved in the production of epidermal hyaluronic acid and contributes to keratinocyte proliferation and differentiation [21]. HBEGF is a major growth factor involved in epithelialization, keratinocyte migration, and skin wound healing [22]. This gene was also prominent in TriHex 1 early studies. SMAD7 is demonstrated to accelerate wound healing through its effects on keratinocyte proliferation and migration and contributes to balancing the wound stroma decreasing hypertrophic scarring [23, 24]. Finally, VEGFA, an angiogenic factor, was identified. Keratinocytes are one of the main sources of VEGF during wound healing by stimulating endothelial cells in blood vessels in the ECM [25] (Table 1).

**TABLE 1:** ScRNA‐sequencing‐differentially expressed genes by cell type.

Gene symbol	Gene name	Cell type	TriHex 1.0 (FC)	TriHex 2.0 (FC)
FST	Follistatin	Keratinocyte	3.7	1.8
HAS3	Hyaluronan synthase 3	Keratinocyte	1.9	1.7
HBEGF	Heparin binding EGF like growth factor	Keratinocyte	3	2.6
SMAD7	SMAD family member 7	Keratinocyte	2.2	1.7
VEGFA	Vascular endothelial growth factor A	Keratinocyte	1.9	2.1
LAMC2	Laminin subunit gamma 2	Keratinocyte	NS	1.7
COL1A2	Collagen type I alpha 2 chain	Keratinocyte	NS	1.5
COL4A1	Collagen type IV alpha 1 chain	Keratinocyte	NS	1.6
LAMB3	Laminin subunit beta 3	Keratinocyte	NS	1.7
LAMC2	Laminin subunit gamma 2	Basal keratinocyte	2.4	1.6
KPRP	Keratinocyte proline rich protein	Basal keratinocyte	3.5	2
CXCL12	C‐X‐C motif chemokine ligand 12	Basal keratinocyte	NS	2.5
BMP4	Bone morphogenetic protein 4	Fibroblast	1.7	1.5
PPARG	Peroxisome proliferator activated receptor gamma	Fibroblast	1.9	1.7
FABP4	Fatty acid binding protein 4	Fibroblast	NS	3.7
COL5A3	Collagen type V alpha 3 chain	Fibroblast	NS	1.7
COL6A3	Collagen type VI alpha 3 chain	Fibroblast	NS	1.7
ITGB3	Integrin subunit beta 3	Fibroblast	NS	1.9
ITGAX	Integrin subunit alpha X	Fibroblast	NS	1.8
TGFB3	Transforming growth factor beta 3	Fibroblast	NS	2
LTBP1	Latent transforming growth factor beta binding protein 1	Fibroblast	NS	1.5
JUN	Jun proto‐oncogene, AP‐1 transcription factor subunit	Fibroblast	NS	−1.6
HIST2H2BE	H2B clustered histone 21	Fibroblast	NS	−1.6

There were 166 keratinocyte upregulated genes unique to TriHex 2.0. A pathway analysis revealed an enrichment for anchoring fibril formation: (LAMC2; ColA‐2; Col IV; LAMB3) Keratinocytes deposit LAMC2 into wound bed at the leading edge. This contributes to angiogenesis, epidermal advancement and wound repair [26]. Human collagen alpha‐2 type I stimulates collagen synthesis, wound healing, and elastin production in normal human dermal fibroblasts [27]. The dermal‐epidermal junction (DEJ) provides the link and continuity between the epidermis and the dermis allowing passage of chemicals, pathogens, water or electrolytes in and out of the body. The major components of this basement membrane are laminins and collagen IV [26, 28]. Human collagen alpha‐2 type I stimulates collagen synthesis, wound healing, and elastin production in normal human dermal fibroblasts [27]. The dermal‐epidermal junction (DEJ) provides the link and continuity between the epidermis and the dermis allowing passage of chemicals, pathogens, water or electrolytes in and out of the body. The major components of this basement membrane are laminins and collagen IV [28]. The *LAMB3* gene is involved in the production of laminin 332 (formerly known as laminin 5) an anchoring filament in the basement membrane. Laminins are proteins that regulate cell growth, movement and adhesion. In addition, they are very involved in the formation of the basement membrane which gives strength and resiliency to the skin. This protein also appears to be important for wound healing [29]. Laminin interactions—LAMC2; Col IVA; LAMB3 (see above) was another enriched pathway among these genes (Table 1).

#### 3.3.2 Basal Keratinocytes

The basal keratinocyte population demonstrated 133 significantly upregulated genes for TriHex 1.0 and 91 for TriHex 2.0. There were 18 genes in common. Among those in common were LAMC2, which is important during skin repair. It helps create a basal membrane for endothelial cells [26]. In addition, KPRP was identified, which is important for barrier function [30]. Seventy‐three genes were significantly upregulated uniquely for TriHex 2.0. Among these, CXCL12, also known as SDF‐1, promotes the recruitment and honing of stem cells which differentiate into endothelial cells and fibroblasts to form the granulation tissue ready for the deposition of collagen. Simultaneously keratinocytes and endothelial cells at the wound edge proliferate to close the wound surface [31] (> 2.0 fold upregulated; Table 1).

#### 3.3.3 Fibroblasts

The fibroblasts revealed 164 significantly upregulated genes by TriHex 1.0 and 144 by TriHex 2.0. Thirty‐nine genes were common to both. Notable among these is BMP4, which is involved in the modulation of cell adhesion, extracellular matrix remodeling, cell motility, and signaling [32]. In addition, PPARG was identified, which is a transcription factor involved in sebocyte and adipocyte differentiation and lipid production. Furthermore, in the skin, it is demonstrated to be essential for maintaining skin barrier permeability [33] (Table 1).

One‐hundred and five upregulated genes were unique to TriHex 2.0. FABP4 demonstrated the largest upregulation. FABP4 is lipid transported that is connected to PPARG and functions to maintain adipogenesis [34]. Importantly, the TriHex 2.0 unique genes were associated with pathways related to ECM remodeling, pre‐conditioning prior to procedures, and anti‐senescence.

ECM sp. organizaiton, specifically collagen formation, was one of the main pathways identified from the TriHex 2.0 unique upregulated genes. Type V collagen (gene COL5A3) was among these. This is an important component of the ECM that interacts with collagen 1 during fiber development [35]. In addition, type VI collagen (gene COL6A3) is a key modulator of ECM synthesis and fibroblast behavior and may play an important role in wound healing and tissue regeneration [36]. Multiple collagens, decorin, integrins, etc. all involved in ECM organization were indentified as significantly upregulated.

Another enriched pathway from the TriHex 2.0 fibroblast genes was molecules associated with elastic fibers. These included integrins (ITGB3 and ITGAX) and TGF‐related genes (TGFB3 and LTBP1). TGF‐β3 plays a role in scar‐free wound healing by preventing the accumulation of unhealthy collagen I and III and boosting fibroblast action during the wound healing process [37].

The fibroblast cell population also demonstrated significantly down regulated genes after TriHex 1.0 and 2.0 treatment. There were 154 significantly down regulated genes with TriHex 1.0 and the same number for TriHex 2.0. Ninety of those genes were common between the two groups. For the 64 unique TriHex 2.0 genes, there was an enrichment of genes associated with the Senescence‐Associated Secretory Phenotype (SASP) pathway. These included JUN and Hist2H2BE. Cellular senescence marks a terminal arrest in fibroblasts and stem cells contributing to aging by inducing the release of inflammatory mediators that are destructive to the ECM milieu. H2B Type 1‐K accumulates in senescent fibroblasts with persistent DNA damage [38] (Table 1).

### 3.4 Ex Vivo Dermoepidermal Junction (DEJ)/Basement Membrane Investigation

**FIGURE 4:**
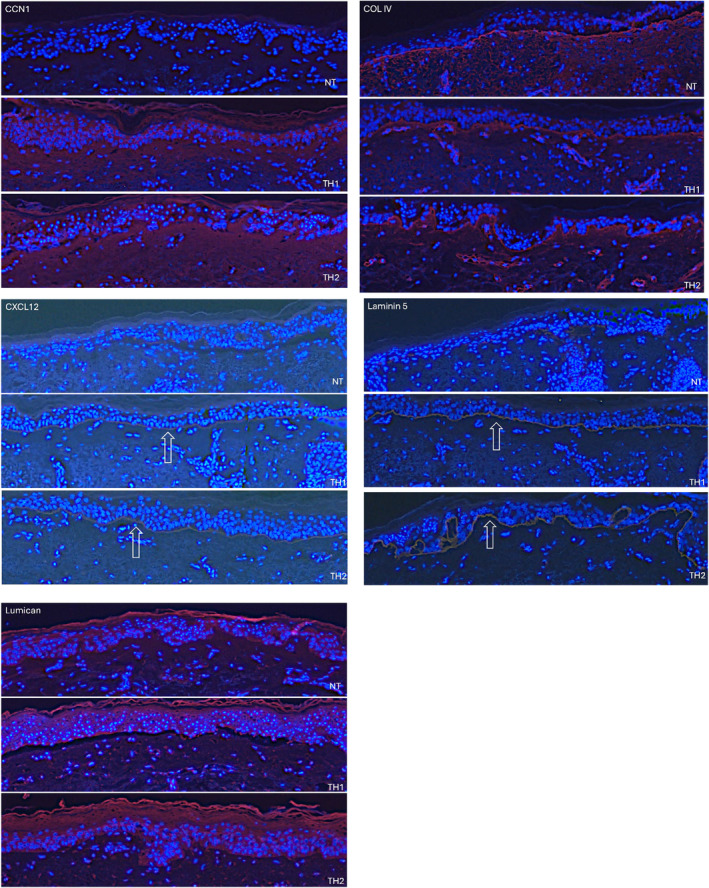
scRNA‐sequencing Validation of TriHex 2.0 Unique Expression. Skin samples were cultured using the established ex vivo model. They were left untreated or treated with TriHex 1.0 (TH1) or TriHex 2.0 (TH2). After 7 days of treatment, the tissue sections were processed for immunostaining to detect CCN1, Col IV, or Lumican in red or CXCL12 or Laminin 5 in yellow (highlighted by the white arrows), and the nuclei are shown in blue.

Based on the identification of hundreds of unique genes identified as being upregulated after TriHex 2.0 as described in section three, CCN1, ColIV, CXCL12, Laminin 5, and Lumican were selected to explore their expression in the ex vivo model after treatment with TriHex 1.0 and TriHex 2.0. CCN1 demonstrated an increased expression near the DEJ after TriHex 1.0 treatment. With TriHex 2.0 treatment, there was an increased expression that expanded beyond the DEJ into the dermis. Col IV expression slightly increased along the DEJ with TriHex 1.0 and was substantially increase after TriHex 2.0. This localization coincides with is role as a basement membrane protein. The expression of CXCL12 was enhanced by TriHex 1.0 and 2.0. However, its expression after TriHex 2.0 treatment revealed a more defined DEJ. Next, Laminin 5 expression increased after TriHex 1.0 and 2.0 treatment. However, after TriHex 2.0 treatment the Laminin 5 expression defined that TriHex 2.0 treated samples yielded refined rete pegs, providing evidence of an improved skin barrier. These data were also enhanced by the expression of Col IV after TriHex 2.0 treatment. Finally, lumican expression was moderately increased in the epidermal region after TriHex 1.0. However, after TriHex 2.0, its expression was further enhanced and showed a distinct increase at the DEJ (Figure 4).

## 4 Discussion and Conclusion

Advancing science usually begins with using current technologies as baseline and seeking improvements and modifications to move the process forward. In this case, TriHex technology has been through multiple validation criteria related to ECM remodeling, optimized healing, and elastin stimulation. Adding a new proprietary peptide to the TriHex combination would need to demonstrate synergistic benefits in these areas and possibly added advantages in related regenerative aspects.

The first exploratory study confirmed new synergies in gene expression. In particular, the powerful up regulation of laminin and its integrin receptor indicated a new basement membrane, dermo epidermal junction (DEJ) activity that is vital to skin health in general. In the aged skin, fragility with loss of membrane thickness and flattening of the rete ridges has been described [39]. Weakened adhesion between dermis and epidermis caused by these DEJ changes, decreases mechanical protection and predispose the skin to tears and fragility [39]. The DEJ provides a regenerative milieu to the keratinocytes encouraging epidermal renewal and promoting skin wound healing [39]. This first study provided a hint that the DEJ could be a good new area of focus with the addition of Octa.

In the second study, the capacity for elastogenesis in an ex vivo model was tested. This is a recurring theme that we have used as researchers in skin technologies—elastin is very representative of regenerative capacity. When we first started exploring the subject in 2015, many of our colleagues argued that elastin could not be regenerated. With multiple biopsies using exclusive stains (Movat etc.) [40] and multiple biopsies in clinical trials we have demonstrated that indeed elastin can be regenerated if the presiding milieu is optimal. To this end, study two demonstrated that MFAP4 (signifying elastin microfibrils) expression increases with TriHex, Octa, and TriHex + Octa treatments. In this case, the increase with TriHex + Octa is demonstrated by an extension of new extended healthy fibrils into the papillary dermis that is greater than TriHex and Octa alone. The expression of MFAP4 indicates that new elastin fibers have a place to form. Additionally, tropoelastin (TE), the core protein molecule of elastin increased with all of the 3—TriHex, Octa, and TriHex + Octa treatments. However, the increase with TriHex + Octa shows a proliferation of new elastin fibers into the papillary dermis that is greater than TriHex and Octa alone. In addition, the alignment of the fibers is more organized with the combination treatment, suggesting that the newly formed elastin fibers are more stable.

In the 3rd validation study, a deeper approach was taken exposing gene upregulation within the different cell populations using single cell gene sequencing analysis. Of particular interest to this segment of the study was keratinocytes (relating to superficial skin health), basal keratinocytes (relating to proliferation and healing capacity) and the fibroblasts (relating to ECM remodeling fibroblast function). Keratinocyte cells showed an anticipated wound healing, ECM remodeling gene response that was common to both groups, TriHex 1 and 2.0 (Follistatin, HAS3, HBEGF, SMAD7, VEGF). Descriptors of these specific gene functions are listed in the results section above. The genes upregulated specific to TriHex 2.0 follow the initial pattern of DEJ strengthening seen in Study one. Thus, laminins and Col IV both integral to DEJ composition were significantly upregulated. The basal keratinocytes showed specific upregulation of CXCL12 (also known as SDF‐1), a gene integral to honing of stem cells, vascularization and wound healing pathways [25]. CCN1 accelerates matrix remodeling and limits fibrosis by turning myofibroblasts into senescent cells, but its primary role is as a critical regulator of wound healing and regeneration [41]. The gene upregulation was then confirmed in the 4th validation study concentrating on the identified genes upregulated by TriHex 2.0 and indeed confirming the presence of these proteins in the ex vivo model with immunofluorescent staining.

Finally, the pathways identified in relation to fibroblast gene interactions, particular to TriHex 2.0 include additional ECM organization and collagen and elastin formation. Of note and particularly relevant was a downregulation pathway identified in the TriHex 2.0 group revolving around cellular senescence and SASP production. Downregulation of these pathways related to fibroblasts is an extremely important strategy that is sought after in new anti‐aging strategies [42, 43, 44].

It is thus apparent that the addition of the Oct‐45 to TriHex technology in the form of TriHex 2.0 is a significant advance to TriHex technology science. This takes the form of:

- Continued ECM remodeling and positive wound healing as with original TriHex.
- Supplementary benefits of added elastin and hyaluronic acid stimulation.
- A new added effect on DEJ/basement membrane with multiple new gene upregulations in this region and in the keratinocyte cells.
- Basal keratinocytes showing additional wound healing gene upregulation.
- Fibroblasts showing ECM remodeling pathways but with additional anti‐senescent effects.

Cumulatively this contributes to continued emphasis on pre‐conditioning of the skin prior to procedures with added components of ECM remodeling, wound healing advantages and significant anti‐aging components involving senescent cell targeting and DEJ strengthening. Clinical studies to follow.

## Author Contributions

A.D.W. developed the science, designed studies, analysis, paper writing. M.E.Z. data analysis, paper writing, study design. F.S. paper writing.

## Conflicts of Interest

Widgerow is Chief Scientific Officer of Galderma, Shafiq is full time employee and Ziegler is part time consultant Alastin, a Galderma company.

## Data Availability

The data that support the findings of this study are available from the corresponding author upon reasonable request.
