# Correction to “TriHex 2.0—Advancing Skin Health Science and the TriHex Technology”

**DOI:** 10.1111/jocd.71122

**Published:** 2026-08-07

A.D. Widgerow , M.E. Ziegler , and F. Shafiq , “TriHex 2.0—Advancing Skin Health Science and the TriHex Technology,” Journal of Cosmetic Dermatology 24, (2025): e16690, 10.1111/jocd.16690.39660586 PMC11845939

In Abstract section, the “Results” subsection, a transcription error was identified where the value “69%” should be updated to be “59%” in the following line:

“HA levels (CD44 intensity) significantly increased with TriHex (69%; *p* < 0.01)”

This should be updated to be as follows:

“HA levels (CD44 intensity) significantly increased with TriHex (59%; *p* < 0.01)”

We apologize for this error.

